# Prevalence of Human Papillomavirus (HPV) in Oesophageal Squamous Cell Carcinoma in Relation to Anatomical Site of the Tumour

**DOI:** 10.1371/journal.pone.0046538

**Published:** 2012-10-12

**Authors:** Hedvig E. Löfdahl, Juan Du, Anders Näsman, Emilia Andersson, Carlos A. Rubio, Yunxia Lu, Torbjörn Ramqvist, Tina Dalianis, Jesper Lagergren, Hanna Dahlstrand

**Affiliations:** 1 Upper Gastrointestinal Research, Department of Molecular Medicine and Surgery, Karolinska Institutet, Stockholm, Sweden; 2 Cancer Center Karolinska, Department of Oncology-Pathology, Karolinska Institutet, Stockholm, Sweden; 3 Division of Cancer Studies, King's College London, London, United Kingdom; Institut Gustave Roussy, France

## Abstract

**Background:**

The prevalence and role of human papillomavirus (HPV) in the aetiology of oesophageal squamous cell carcinoma is uncertain. Based on the presence of HPV in the oral cavity and its causal association with squamous cell carcinoma of the oropharynx, we hypothesised that HPV is more strongly associated with proximal than distal oesophageal squamous cell carcinoma.

**Methods:**

A population-based study comparing HPV infection in relation to tumour site in patients diagnosed with oesophageal squamous cell carcinomas in the Stockholm County in 1999–2006. Multiplex polymerase chain reaction genotyping (PCR) with Luminex was conducted on pre-treatment endoscopic biopsies to identify type specify HPV. Carcinogenic activity of HPV was assessed by p16^INK4a^ expression. Multivariable logistic regression was used to calculate odds ratios and 95% confidence intervals.

**Results:**

Among 204 patients, 20 (10%) had tumours harbouring HPV DNA, almost all (90%) of HPV high-risk type, mainly HPV16. Tumours containing HPV were not overrepresented in the upper compared to the middle or lower third of the oesophagus (odds ratio 0.6, 95% confidence interval 0.2–1.9). P16^INK4a^ expression was similarly common (24% and 16%) in the HPV-positive and HPV-negative groups.

**Conclusion:**

This study found a limited presence of HPV in oesophageal squamous cell carcinoma of uncertain oncogenic relevance and did not demonstrate that HPV was more strongly associated with proximal than distal tumours.

## Introduction

The introduction of preventive vaccines against human papillomavirus (HPV) has increased the incentive to clarify possible causal links between HPV and non-cervical carcinomas, for example oesophageal squamous cell carcinoma cancer. Oesophageal cancer is the 8th most common type of cancer and the 6th most common cause of death from cancer, globally, [1] and the 5-year survival is only about 10% in Western countries. [2] Therefore, preventive measures are highly warranted, and HPV infection might be one such target. HPV is causally linked with cervical cancer, [3], [4], [5] and has recently also been established as a risk factor for oropharyngeal squamous cell carcinoma. [6], [7], [8], [9] An association between HPV and oesophageal squamous cell carcinoma was initially suggested in 1982 when morphological similarities between HPV induced lesions in the genital tract and oesophageal squamous cell carcinomas were found. [10] Since then several studies have been conducted, using varying or no control subjects and different virus detection methods, producing conflicting results. The frequency of HPV DNA in oesophageal squamous cell carcinomas shows a great diversity in frequency around the world, with usually high HPV incidence in high-risk areas including China, [11] although recently contradicted [12] while studies from North America and Europe indicate a lower frequency (0–30%). [13], [14], [15], [16], [17] It has also been reported that patients with current or a history of HPV positive head and neck cancer also have an increased risk of HPV related squamous cell cancer in the oesophagus. [18] Because of the association of HPV with oropharyngeal cancer, the presence of HPV in the oral cavity, and the varying results in previous research, we hypothesised that HPV would be associated with a more proximal than distal site in oesophageal squamous cell carcinoma. The present study aimed to test this hypothesis.

## Materials and Methods

### Study design

This population-based study included patients diagnosed with oesophageal squamous cell carcinoma in the Stockholm County in Sweden during the period 1999 to 2006. The patients were identified through the Swedish Cancer Register, a nationwide register initiated in 1958 with a completeness of 96% for all cancers [19] and 98% for oesophageal cancer. [20] Medical records were examined to enable categorisation of patients into 3 groups depending on tumour localisation: upper (15–24 centimetres from the tooth line), middle (24–32 centimetres from the tooth line), or lower (32–40 centimetres from the tooth line) third of the oesophagus. Histologically confirmed paraffin embedded endoscopic biopsies from oesophageal squamous cell carcinomas, obtained before treatment, were reviewed by one pathologist (CL). To avoid detection bias, only endoscopic biopsies were included. Surgical resection is more common in distal compared to proximal oesophageal tumours, and analysis of resected tissue could provide a better assessment of HPV infection, and the detection of HPV between the locations of the tumours could be biased if surgical specimens would have been included.

### Detection of human papilloma virus

Detection of HPV in the tumour biopsies was conducted as follows. Formalin-fixated paraffin embedded biopsies were sectioned into 4×15 µm slices on glass, and macro-dissected to make sure that the section contained at least 70% tumour cells. To minimise contamination, blanks were added after every 5th sample and treated in the same way as regular samples during slicing and micro-dissection. DNA extraction was performed using the High Pure RNA Paraffin Kit (Roche's) without DNAse. PCR was performed by adding 10 µl of DNA sample and 40 µl of reaction mixture, containing broad-spectrum GP5+/6+ primers (BGP5+/BGP6+), to the Qiagen Multiplex PCR Master Mix (Qiagen, Hilden, Germany) [21]. Amplification was made in 40 cycles, with an annealing temperature of 38°C for 90 seconds. HPV genotyping was conducted with Luminex [22], [23] together with a Multimetrix kit (Heidelberg, Germany), analysing 24 HPV types, including 15 HR types (16, 18, 31, 33, 35, 39, 45, 51, 52, 56, 58, 59, 68, 73, 82), 3 putative HR types (26, 53, 66) and 6 LR types (6, 11, 42, 43, 44, 70) [24]. Hybridisation of PCR products (10 µl) and Luminex beads (40 µl) together with the subsequent steps were performed using standard methods. The samples were analysed using a Luminex 100 analyser (BioRad Laboratories, Hercules, USA) with the cut-off limits proposed by Multimetrix.

### Detection of p16^INK4a^ by immunohistochemistry

To assess biological activity of HPV in the tumours, p16^INK4a^, a surrogate marker for HPV activity, [25], [26] was measured with immunohistochemistry. The biopsies were sectioned and stained using the monoclonal antibody p16 ^INK4a^ (delusion 1∶200, clone JC8, Santa Cruz Biotechnology, INC) and a standard streptavidin-biotin peroxidase method. One pathologist (EA) examined and graded all samples according to predetermined conditions, and samples were categorised as negative if no of the tumour cells were staining for p16 ^INK4a^, weakly and unspecifically stained if a minority and ungrouped tumour cells were stained, and positive if there was a homogeneous staining of a clear majority of the tumour cells were stained for p16 ^INK4a^. The slides showing weak and unspecific staining were included in the p16 ^INK4a^ negative category in the analysis.

### Statistical analysis

Fisher's exact test was used to identify predictors for HPV-positive tumours. A p-value below 0.05 was considered statistically significant. Unconditional logistic regression was used to calculate relative risk between HPV infection and risk of various tumour sites in the oesophagus, expressed as odds ratios (OR) with 95% confidence intervals (CI). Using multivariable modelling, ORs were adjusted for age (grouped into <60, 60–70, or >70 years), sex, and tumour differentiation (high, middle, or low). Any missing data was grouped into a separate category in these analyses.

### Ethics

The study was approved by the Regional Ethics Review Board in Stockholm.

## Results

### Study participants

Among 348 new cases of oesophageal squamous cell carcinoma in the Stockholm County between 1999 and 2006, 67 (19%) were excluded because of; tumour misclassification (n = 3, 1%), diagnosis based on autopsy results (n = 11, 3%), or failure to identify any endoscopic biopsy (n = 53, 15%). Among 281 (81%) eligible patients, 77 (27%) were classified as non-participants; 51 (18%) because of lack of tumour material and 26 (9%) because of tumour material being unavailable. Finally, 204 patients (73%) remained for the present study. Characteristics of the participants, non-participants and excluded patients are presented in Table 1. The distribution of sex, age and tumour location were similar between these groups, except for more missing data regarding tumour location in the excluded group (Table 1).

**Table 1:** Differences between included and excluded patients.

Variable	Participants	Non-participants/excluded*
	Number (%)	Number (%)
**Total**	204 (59)	144 (41)
**Sex**		
Men	127 (62)	94 (65)
Women	77 (38)	50 (35)
**Age at diagnosis (years)**		
<60	37 (18)	23 (16)
60–70	76 (37)	52 (36)
>70	91 (45)	69 (48)
**Tumour location** ^#^		
Upper third	53 (26)	23 (16)
Middle third	64 (31)	45 (31)
Lower third	83 (41)	45 (31)
Unspecified	2 (1)	5 (4)
Missing	2 (1)	26 (18)

* Nonparticipants include low DNA level (n = 51, 18%) and unable to collect the endoscopic biopsy (n = 26, 9%). Excluded participants include tumour misclassification (n = 3, 1%), tumour detected at autopsy (n = 11, 3%) and unavailable endoscopic material (n = 53, 15%).

#Tumour location was similar in the participants and non-participant/excluded groups (p = 0.113, Fisheŕs exact test) except for more missing in the non-participant/excluded group p<0.001, Fisheŕs exact test).

### Detection of HPV and p16^INK4a^

Characteristics of patients with HPV-positive and HPV-negative tumours are presented in Table 2. In total, 20 (10%) tumours were positive for HPV DNA, of which 18 (90%) harboured high-risk HPV types (16 (n = 13), 33 (n = 1), 45 (n = 1), 51 (n = 2), 52 (n = 1)1, 73 (n = 1), 82 (n = 1)1, 1 (5%) putative high-risk type (66)1 and 1 (5%) low-risk type (42). P16***^INK4a^*** expression was evaluated in 17/20 (85%) HPV positive cases (3 lacked any material left) and in 113 HPV negative cases (6,5 HPV negative control per HPV positive case). The prevalence of p16***^INK4a^*** expression in the HPV-positive (24%) and HPV-negative (16%) tumours was not statistically significantly different (Table 2).

**Table 2:** Characteristics of HPV positive versus HPV negative participants.

Variable	HPV negative	HPV positive	p-value
	Number (%)	Number (%)	
**Total**	184 (90)	20 (10)	
**Sex**			
Men	113 (61)	14 (70)	0.628
Women	71 (39)	6 (30)	
**Age at diagnosis (years)**			
<60	32 (17)	5 (25)	0.426
60–70	71 (39)	5 (25)	
>70	81 (44)	10 (50)	
**Tumor location**			
Upper third	49 (27)	4 (20)	0.151
Middle third	53 (29)	11 (55)	
Lower third	78 (42)	5 (25)	
**Tumor differentiation**			
High	10 (5)	2 (9)	0.426
Middle	100 (54)	13 (62)	
Low	72 (39)	6 (29)	
**Total** *	113 (87)	17 (13)	
**p16**			
Negative	95 (84)	13 (76)	0.488
Positive	18 (16)	4 (24)	

* p16 analysis was conducted on 130 (64%) out of 204 patients.

Percentages not adding to 100% are due to missing data.

### Association between HPV and tumour site

Patients with tumours containing HPV DNA did not have any increased risk of having a tumour located in the upper third of the oesophagus compared to a more distal site (adjusted OR 0.6, 95% CI 0.2–1.9, Table 3). The comparison of upper/middle versus lower tumour site rendered an adjusted OR of 2.1 (95% CI 0.7–6.3, Table 3). The majority of the HPV-positive tumours were rather located in the middle oesophagus (55), and the HPV-negative tumours were more frequently found in the distal oesophagus (42%), although no statistically significant differences were observed.

**Table 3:** Risk of different sites for oesophageal squamous cell carcinoma when exposed to HPV, expressed as odds ratios (OR) with 95% confidence intervals (CI).

Variable	Upper	Middle/lower	Crude model	Adjusted model
	Number (%)	Number (%)	OR 95% CI*	OR 95% CI^#^
**Total**	53 (27)	147 (74)		
**HPV status**				
Negative	49 (92)	131 (89)	Reference	Reference
Positive	4 (8)	16 (11)	0.7 (0.2–2.1)	0.6 (0.2–1.9)

Variable	Upper/middle	Lower	Crude model	Adjusted model
	Number (%)	Number (%)	OR 95% CI*	OR 95% CI^#^
**Total**	117 (59)	83 (42)		
**HPV status**				
Negative	102 (87)	78 (94)	Reference	Reference
Positive	15 (13)	5 (6)	2.2 (0.8–6.4)	2.1 (0.7–6.3)

* No adjustments made.

# Adjustments made for sex, age and tumour differentiation.

## Discussion

This study indicates a limited presence of human papillomavirus (HPV) DNA in oesophageal squamous cell carcinoma in a Swedish population, and does not provide support for the hypothesis of an increased prevalence of squamous cell carcinomas infected with HPV in the proximal compared to the distal oesophagus. Furthermore, the p16^INK4a^ data do not support a carcinogenetic role in the oesophageal squamous cell cancers harbouring high-risk HPV.

Strengths of the present study include its population-based design, the high number of patients included compared to other studies in the same area, the valid assessment of the exposure and outcome, and the ability to adjust for potential confounding factors. The use of oesophageal squamous cell carcinoma patients as both case and control subjects ensured similar risk factor profiles, such as for example smoking and alcohol use, and thus reduced confounding. The use of endoscopic biopsies in all participants circumvented biased detection of HPV infection between tumour sites. The biopsies were taken prior to any preoperative treatment, which ensured that the studied tumour material was not affected by e.g. chemotherapy or radiotherapy. Finally, the detection of HPV DNA using Luminex on PCR product is an established method with a high sensitivity. [22]

Limitations include a low statistical power, which was largely due to insufficient DNA material in some biopsies and a low prevalence of HPV infection in the tumours. Furthermore, due to the limited size of the tumour biopsies, we could not assay for HPV E6 and E7 mRNA as marker for active HPV in tumour cells, instead we analysed for p16 ^INK4a^, which has previously been used as a surrogate marker for HPV in cervical cancer [27] and HPV activity in head and neck cancer. However, the small endoscopic biopsies may have lessened the reliability of the p16^INK4a^ immunohistochemistry. Another concern was selection bias introduced by patients from certain hospitals more often being excluded.

This study did not reveal any higher occurrence of HPV infection in the tumour location of the upper third of the oesophagus as hypothesised. Instead, most tumours positive for HPV DNA were located in the middle third of the oesophagus. This might merely be an effect of chance, or speculatively this might be a true finding. The middle part of the oesophagus, the transition zone, holds no significant contraction amplitude when measuring peristaltic contractions, [28] and this region is collapsed in resting state, which might allow virus particles to implant more easily.

To our knowledge, this is the first study comparing the association between HPV infection and the development of squamous cell carcinoma in different locations in the oesophagus as the primary endpoint. Previous studies show diversity in frequency of tumours containing HPV DNA. A review article from 2002 looking at frequency of HPV DNA in oesophageal tumours showed great diversity of between 0 and 70% of tumours containing HPV DNA. [11] Different methods were used for detection of HPV, however when pooling the studies using only PCR, the incidence of HPV was 15% (308/2020), which is comparable to the present study (10%). Our results are also consistent with a previous study from Sweden looking at HPV in oesophageal squamous cell carcinoma, where 16% of tumours contained HPV DNA. [14]

Although 90% of the HPV positive tumours harboured high-risk HPV types (and 5% putative high-risk types), we found no correlation between the subset of tumours containing HPV DNA and p16 ^INK4a^ expression. The low portion (24%) of overexpression of p16^INK4a^ in the HPV positive cancer biopsies indicate that HPV may not be involved in tumour progression in oesophageal cancer to a large extent. These results are in line with a recent case-control study of 222 patients with oesophageal squamous cell carcinoma. [17] Only 3.6% (8/222) of tumours were HPV positive and only 4 of the 8 HPV positive cases also overexpressed p16 ^INK4a^. [17] However, it is not implausible that the small endoscopic biopsies lessened the reliability of immunohistochemistry.

In conclusion, this population-based study from Sweden found limited presence of HPV with uncertain oncogenic relevance in oesophageal squamous cell carcinoma and could not demonstrate that HPV was more likely associated to proximal than distal squamous cell carcinomas of the oesophagus.

### Novelty and impact statement

The presence of human papillomavirus (HPV) in oesophageal squamous cell carcinoma was not correlated to the tumour location within the oesophagus
